# An Electrical "Hospital"??

**Published:** 1898-04-16

**Authors:** 


### AN ELECTRICAL "HOSPITAL"? ?

At the Kensington Town Hall, on Monday evening, Mr. C. Luxmoore Drew held an inquiry touching the death of John Slater, aged 71 years, an artist, who was found dead in his room at 51, Peel Street, Kensington, on Good Friday. He had been going through a course of electrical treatment at the Notting Hill Gate Free Hospital.

The Coroner said the deceased had been receiving treatment by electricity at a so-called hospital, but on his death the people there were unable to give a certificate as to the cause of death. It was a serious matter when people without proper skill and knowledge treated a person, because they might aggravate the disease and accelerate the death. In some cases electricity was beneficial, but in most hospitals there was a special department for it, and he could not understand why the deceased did not ask for treatment at St. Mary's Hospital instead of going to a place outside.

The brother of the deceased said he came to London some time since to follow his occupation and to get advice. He complained of a feeble heart. He wrote home that he was undergoing a course of electrical treatment, and, when urged, refused to return home. He had no means beyond his earnings, and on several occasions witness sent him money, the last being £2 a week before his death.

Elizabeth Smith, the landlady of the deceased at 51, Peel Street, said the deceased had been there two months. He was not in good health, complaining of lameness and shortness of breath. Witness knew he was attending a place at Silver Street where the battery was applied, but he did not seem to improve. She understood he paid a guinea a week for treatment. He did not do much work, and she tried to advise him to go to a proper hospital, but he said he knew which did him the most good. He was not particularly clean in his habits. On Good Friday morning she found him in his room, sitting in a chair fully dressed, quite dead. She sent to the "Professor" at the hospital where the deceased had been attending, but he said he did not go to patients outside. There was a slight injury to his face, which was caused by a fall a few days previously in his room when alone.

Thomas Moss, Coroner's officer, deposed to searching the room, and finding 9s. 11½d., letters, and the inhaler produced. He called at the institution at 30, Silver Street, Notting Hill Gate, Kensington, and saw the "Professor." He said the deceased had rheumatism, and he thought a weak heart. The deceased had paid £3. The deceased was rather dirty in his habits.

Annie Bridge, the next witness, said she was a nurse at 30, Silver Street, which was the "electrical hospital."

In reply to the Coroner, the witness said the name of the "institution" was the Notting Hill Gate Free Hospital. There were six nurses there, and the medical staff consisted of Dr. Dalton, the consulting physician, who lived at Swanage, and did not see any patients. Professor D'Odiardi treated the patients for rheumatism.

For chronic cases and incurable cases?—Yes.

Consumption, throat disease, locomotor ataxy, paralysis, blindness, long and short sight, and deafness; debility, fagged brains, internal diseases and tumours, uterine affections, dyspepsia, influenza, and special treatment of the voice for public singers, speakers, &c.?—Yes, sir. (A laugh.)

And nurses are sent into the country with the X Rays?—Yes, sir.

Witness explained that they did not take photographs of persons, but simply directed the Rays on the spot of the disease. They were used in cases of people who are blind. Witness had had no hospital training except under the professor for four years. Deceased was given electricity under the direction of the professor. He gave £3 voluntarily to the institution, and was not asked for it. The institution was kept going by the professor, and not by voluntary contributions. There was a board of management, but she did not know if they subscribed. She was paid by the committee. Those of the patients who could afford it, paid, while the others who could not, came free. The battery was applied to deceased for rheumatism and a bad leg, and he said he was better afterwards.

The Coroner remarked that electricity ought to be given with great care, and only under the direction of a doctor. (The Jury: "Hear, hear.")

Edmond Savaly D'Odiardi, of the Notting Hill Gate Free Hospital, was the next witness. He gave his evidence while labouring under great mental excitement. Speaking in broken English, his general answer to any question was to burst out with an address on the benefits of his electrical treatment, the volubility with which it was uttered making his words almost indistinguishable. Eventually the Coroner had to ask him to kindly answer Yes or No, and not to fence with the questions in the way he was doing. The jury also expressed their disapproval of the manner in which the "professor" tried to avoid answering pointed questions. It was eventually extracted from him that he was a medical electrician, but had no medical training or qualification. He had never gone through a course of studies, and had never seen a post-mortem. Asked if he had ever seen a tumour in a human body, witness replied that he had seen them in schools of anatomy at hospitals. He came of a French, German, and English family, and had worked under many well-known doctors. He confined himself to electrical science, and his advice had been accepted and his instruments had been used by the Dean of the Medical Faculty of Belgium in 1889. He used it to affect the physical forces of the human body. He had benefitted the voice of clergymen, barristers, and singers.

The Coroner: Like doctors sending masseurs, only others give it haphazard, and you give it scientifically.

Witness said he founded the hospital with Judge Pennington and Lady Forsyth, and it had already cost more than £2,000 above the contributions received. Witness was the medical electrician. A short time ago General MacLaughlin was appointed chairman, and up to the present they had worked with the doctors who had sent them patients. Witness was the main mover in the hospital, and they had no medical staff. The institution was supported for five years by Judge Pennington, and since that time his friends who had attended the hospital had paid. Patients paid what they liked, and the staff were paid out of it. Deceased was treated for muscular paralysis and numbness of the legs. He got a cold after, and promised to go to a hospital with it.

The Coroner said electricity might affect a person with a weak heart.—The Witness replied that the battery was never applied to the heart, but only to the lower extremities, and then only in a system of weak currents. There was no danger whatever in giving a person with a weak heart electricity.

You give it without medical advice?—No. I always ask for their doctor.

Did the deceased's physician advise it in his case.—No.

Did you ask for his physician, even?—No, sir.

The Coroner said it would be a serious thing if a person died under his treatment, and that the death was hastened in any way by the treatment.—Witness added that, in his opinion, the deceased was suffering from catarrh of the stomach. The throat also was congested; and he thought he had a weak heart. Witness did not examine his chest.

The Coroner advised the witness for his own sake in future not to give the battery except under a doctor's advice. They would hear from the doctor whether the treatment was correct and beneficial; but it appeared the deceased had been given it not only for the rheumatism, but also for dyspepsia, the kidneys, and bladder.

The Witness proceeded to argue with the Coroner on a point of medical knowledge.

The Coroner: I cannot have that. You must not argue. I cannot ask the jury to take a statement of the kind from a person who has no medical knowledge.

In reply to further questions, Witness said he treated almost any disease. He had been very successful in cases of consumption.

You also send out the X Rays?—Yes; it is very mild, and I have applied it to no less than nine members of a distinguished Cabinet Minister's family—Mr. Chamberlain's.

The Coroner: You ought not to mention private persons' names in that way unless you are asked. Why do you use it?—To reanimate capillary circulation. We find it very useful in many diseases which cause a weak circulation.

Are you connected with an institution in Oxford Street?—No, sir.

Dr. Barratt, of 2, Peel Place, said he was called after death, and had made a post mortem. He found the heart considerably diseased, with old pericarditis and valvular mischief. There was thickening of the mitral valve, and a clot the size of a hen's egg. There was also dilatation, but the aorta was intact. It was a heart that might fail at any moment. There was no catarrh in the stomach, and witness had never heard of a connection between that and a weak heart. Death was due to syncope from heart disease, accelerated by the old pericarditis. He could not say if there had been any previous shock, but it would be a risky thing to give electricity to a heart in that condition. A battery ought never to be applied, except under a doctor's directions. He certainly should not have allowed it to be given to deceased, because it might have benefited his bad leg, but certainly not his heart and kidneys. It would also be a risky thing to give in cases of consumption.

The Coroner explained to the jury that the professor, being unqualified, was absolutely incompetent to diagnose disease. In this case he had treated for a weak heart when it was badly diseased. He had only diagnosed catarrh of the stomach, and that the doctor found did not exist at all. If treatment by such a man accelerated the death, he would be held guilty of manslaughter, or if deceased had died while electricity was being given a charge of manslaughter would have followed. He would advise the witness only to give the battery under a doctor's direction. This death, fortunately, was not connected with the treatment received at this so-called hospital; but if the jury thought there had been neglect and carelessness in giving it to a man in the deceased's condition, they could pass any remark they wished.

After some deliberation in private the jury agreed that the death was due to natural causes, but they added a rider that they considered the hospital people should only apply the battery under medical advice, and that only nurses who had had a thorough hospital training should be engaged.

The Coroner quite agreed with the verdict and rider. He could not understand why people went to such places when they could get the best treatment and care at a proper hospital. Addressing the professor, the Coroner told him it was his duty to warn him that had the man died under his treatment, he would have found himself in a most serious position. After this caution, however, it placed him in a still more serious position, because if death occurred after the application of the battery, without supervision of a medical man and without proper diagnosis that he was a fit subject, he would be held directly responsible. He advised him for his own sake never to attempt to diagnose an internal complaint, or to apply the battery except under a doctor's advice.

The Professor remarked that he considered it very harsh and unjust, and then left the Court.

